# Kikuchi-Fujimoto Disease Associated with Symptomatic CD4 Lymphocytopenia

**DOI:** 10.1155/2014/768321

**Published:** 2014-09-17

**Authors:** Meera Yogarajah, Bhradeev Sivasambu

**Affiliations:** Department of Medicine, Interfaith Medical Center, 1545 Atlantic Avenue, Brooklyn, NY 11213, USA

## Abstract

Kikuchi-Fujimoto disease is a rare benign condition of necrotising histiocytic lymphadenitis with unknown aetiology. We describe here a 30-year-old African American female who presented with fever, generalized rash, cervical lymphadenopathy, and oral candidiasis and was found to have Kikuchi-Fujimoto disease on lymph node biopsy with low CD4 count. The initial presentation was concerning for acute retroviral infection. Her HIV serology and HIV RNA PCR were negative however she had low CD4 count with reversal of CD4/CD8 ratio. Although low CD4 count has been associated with autoimmune disease, it has not been described with Kikuchi-Fujimoto disease. We report the first case of Kikuchi-Fujimoto disease associated with symptomatic CD4 lymphocytopenia.

## 1. Introduction

Kikuchi-Fujimoto disease was first described in 1972 in Japan. Histopathology of the involved lymph nodes differentiates Kikuchi-Fujimoto disease from other lymphadenopathy. Idiopathic CD4+ T cell lymphocytopenia was recognized during HIV epidemic. Some patients suspected of having HIV due to severe CD4+ lymphocytopenia had no evidence of HIV infection. Kikuchi-Fujimoto disease and CD4 lymphocytopenia are rare clinical entities on their own and their pathogenesis is not fully understood; hence, the coexistence of both together in this case makes it significant.

## 2. Case Report

**Figure 1:**
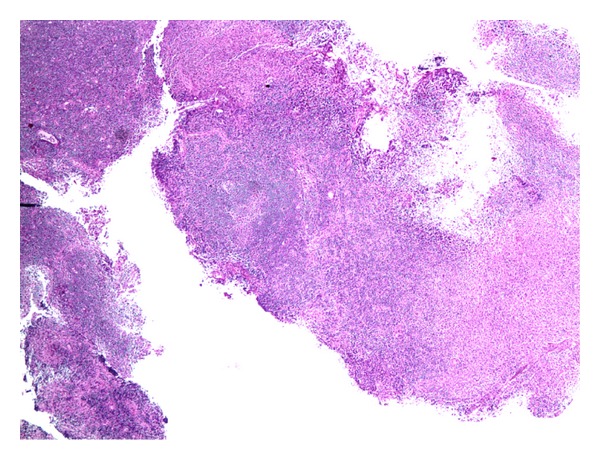
Low magnification-lymph node with focal well circumscribed paracortical necrotizing lesions.

**Figure 2:**
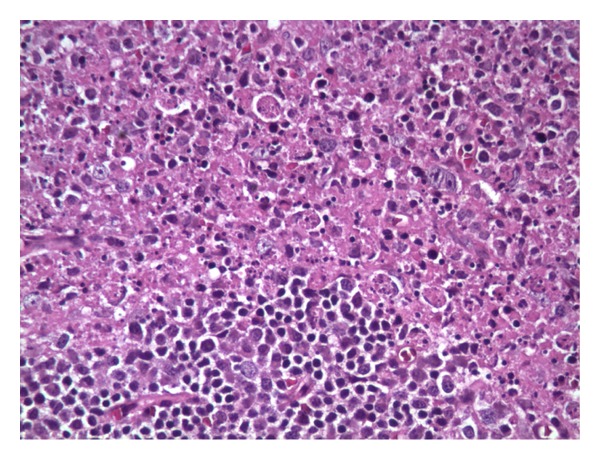
High magnification-lymph node with abundant karyorrhectic debris with scattered fibrin deposits and large mononuclear cells.

30-year-old African American female presented with fever, generalized rash, and occipital headache for 3-day duration. Rash was not pruritic or painful. She had also noticed a mass in the left side of the neck for the last 2 months which was not painful and not progressively increasing in size and was treated with antibiotics repeatedly with no improvement. On examination, she was found to have a temperature of 103, oral thrush, nontender bilateral cervical lymphadenopathy, generalized erythematous, and nonblanching macular papular rash. There was no neck rigidity or hepatosplenomegaly. Labs on admission revealed pancytopenia and elevated ESR. Spinal tap was done and meningitis was ruled out as patient had headache with fever. CT of head was normal. CT of chest abdomen pelvis was done to assess the extent of lymphadenopathy and did not show any other lymphadenopathy or hepatosplenomegaly. Immunologic workup and infectious workup including HIV were sent. ANA was positive with mildly elevated anti-RNP antibody; however, DSDNA and anti-Smith antibody were negative. EBV, CMV,* Ehrlichia*, and Rocky Mountain spotted fever (RMSF) antibodies were negative. HIV antibody and HIV RNA PCR were negative. However, patient was found to have a low CD4 count of 205 with reversal of CD4 to CD8 ratio (0.86). Lymph node biopsy showed necrotising histiocytic lymphadenitis (Figures 1 and 2) diagnostic of Kikuchi-Fujimoto disease. This is one of the possibilities for the clinical syndrome the patient depicted with HIV, lymphoma, and systemic lupus erythematosus (SLE), being other possibilities. This patient presented with Kikuchi-Fujimoto disease associated with symptomatic CD4 lymphocytopenia.

## 3. Discussion

Kikuchi-Fujimoto disease also called Kikuchi disease or Kikuchi histiocytic necrotizing lymphadenitis [1–3] was originally described in young women and is a rare, benign condition of unknown etiology usually characterized by cervical lymphadenopathy, fever [4], and rash [5]. Histopathology of the involved lymph nodes differentiates Kikuchi-Fujimoto disease from several more serious conditions such as acute retroviral infection, lymphoma, and SLE. Kikuchi-Fujimoto disease is a clinical entity to be considered in any patient presenting with fever cervical lymphadenopathy and rash as Kikuchi disease is a self-limiting illness [4]. Awareness of this disorder will help prevent misdiagnosis and inappropriate treatment.

The pathogenesis of Kikuchi disease is unknown. An immune response of T cells and histiocytes to an infectious agent is postulated. Parvovirus B19 [6, 7], Epstein-Barr virus [7, 8], Human Herpesvirus 6, Human Herpesvirus 8, Human immunodeficiency virus, paramyxoviruses, parainfluenza virus,* Yersinia enterocolitica*, and Toxoplasma have been implicated in previous studies.

Kikuchi-Fujimoto disease shares sex and age predisposition as well as histologic features with systemic lupus erythematosus. While initially described in young women, Kikuchi disease also occurs in men. It was initially described in two cases from Japan. However, it has been found in all racial and ethnic groups in most of the countries. Most of the patients were Whites in the United States [1].

The diagnosis of Kikuchi disease is made by lymph node biopsy. The histology of the lymph node in Kikuchi disease may be similar to patients with systemic lupus erythematosus. However, it can be differentiated from infectious conditions presenting similarly. Microscopic examination usually shows single or multiple paracortical foci often with necrosis and a histiocytic cellular infiltrate. Capsule may be infiltrated and perinodal inflammation is seen frequently [9, 10].

Our patient was diagnosed with Kikuchi based on lymph node biopsy. We are reporting this rare case of Kikuchi associated with symptomatic CD4 lymphocytopenia.

Idiopathic CD4+ T cell lymphocytopenia (ICL) was recognized during HIV epidemic. Some patients suspected of having HIV due to severe CD4+ lymphocytopenia had no evidence of HIV infection as in our patient in whom both HIV antibody and PCR were negative [11, 12].

There has been no known causal relationship or association demonstrated to date with regard to CD4 lymphocytopenia and Kikuchi disease. However, CD4 lymphocytopenia is associated with autoimmune disease [13]. Given a possible autoimmune etiology of Kikuchi disease, the question is whether Kikuchi disease leads to CD4 lymphocytopenia. Though many possible etiologies have been postulated for CD4 lymphocytopenia so far there have been no reports with regard to Kikuchi disease as a possible etiology.

Here, we present the first case report of Kikuchi-Fujimoto disease associated with CD4 lymphocytopenia. To conclude, this case emphasizes the importance of considering Kikuchi-Fujimoto disease in patients with idiopathic CD4 lymphocytopenia.
